# Evaluation of the distortion of photographs using various focal lengths

**DOI:** 10.6026/97320630017814

**Published:** 2021-09-30

**Authors:** Nilesh Suresh, Arvind Sivakumar

**Affiliations:** 1 Department of Orthodontics and Dentofacial Orthopaedics, Saveetha Dental College and Hospital, Saveetha Institute of Medical and Technical Sciences, Saveetha University, Chennai, India

**Keywords:** Orthodontics, photography, distortion, survey

## Abstract

It is of interest to evaluate the distortion of intraoral frontal photographs using various focal lengths. Intra-oral frontal view pictures of 20 subjects were taken using a standard 18-55mm lens of a DSLR camera at three different focal lengths (18mm, 35mm, 55mm). 52 experienced orthodontists were chosen and they were asked to assess the photographs and choose the one with the least amount of distortion. Majority of the orthodontists selected the frontal photograph taken at a focal length of 35mm as the best photo with the least distortion among the three photographs. The photos taken at a focal length of 35 mm showed the least amount of distortion.

## Background:

Digital intraoral photography has greatly influenced the ease of documentation and storage of clinical images at different stages. [1] There are numerous applications for digital photography in dentistry which include diagnosis and treatment planning in which the digital photographs are an invaluable diagnostic tool. It provides the clinician, specialist, and technician with an instant visualization of the clinical setting without the need for the presence of the patient and also helps in treatment planning. Pre and post treatment photographs are legal documents, which ideally have to be taken for every patient. [2] Identification of human remains and the analysis of dental-related trauma (ie, human bite marks) through digital photographs can provide accurate reproduction of detail for forensic purposes. [3] A series of photographic images of previous treatment accomplished with other patients can provide a detailed explanation of a specific dental procedure and treatment alternatives. [1] This also helps in motivating the patient. The other essential advantages of digital photography are the ease of transmitting the images, opportunity of detailed editing, and ease of archiving. Periodic digital photographs of a patient's clinical condition can provide immediate visual illustration of improvement or progression of a disease process (ie, caries, gingivitis, periodontitis).[3,4] In addition, photographic series can be used to describe a clinical condition or to communicate ideas and concepts with colleagues in lecture presentations, publications, and professional certification. Distortion free photographs are an essential requirement for using these photographs for the above purposes. An ideal intraoral photograph must have an adequate coverage of the structures with proper angulation, proper lighting without over or under exposure and a good colour reproduction with a proper white balance. There must be no shadows and must capture a crisp, sharp, well-focused image of all the structures. [5] Good quality retractors and mirrors must be used to retract and expose the necessary structures. [6] One of the commonest errors, which most beginners make while taking photographs, is the optical distortion caused due to the wide-angle lenses called the barrel distortion (Figure 1 - see PDF). [5] A wide-angle lens requires a close subject to fill the field and can result in "barrel distortion", involving enlargement of the chin and nose, elongation in the anteroposterior dimension, and excessive curvature on the sides. An extremely powerful telephoto lens creates "compression distortion", with nearer objects appearing smaller, shortening in the anteroposterior dimension, and excessive flattening of features. [7] Therefore, it is of interest to evaluate the barrel distortion seen while taking an intraoral frontal photograph at three different focal lengths (18mm, 35mm & 55mm) and to find the focal length, which produces least distortion.

## Materials and Methodology:

This was a prospective cross-sectional photographic questionnaire study. A set of three intra-oral frontal photographs, each at focal length of 18mm, 35mm and 55mm were taken using a standard EF-S18-55 IS STM lens on a Canon 200D DSLR camera (Canon EOS Rebel SL2) with a ring flash (Yongnuo YN-14EX TTL LED) for twenty subjects. The camera was held at the same angulation but the distance between the lens and the subject was altered according to the three different focal lengths settings. A total of 60 intra oral frontal photographs were taken. The point of focus for all the 60 images were at the premolar region in orders to get all the teeth in focus. The subjects included in the study were patients who had completed their orthodontic treatment with good alignment of teeth and had no missing teeth (excluding the third molars). Subjects with gross asymmetries were excluded from the study.  All the photos were taken using the same camera settings by a single experienced operator. The shutter speed was set at 1/40 secs, with aperture size set at f 22 and ISO of 400. An extraoral smile photo of the patient was additionally taken for all the 20 subjects using standard settings of shutter speed of 1/40 sec, aperture size of f10 and ISO of 400 with two softboxes on either side of the camera to avoid shadow. This was taken as a reference photograph to check for distortion of intraoral photographs. All the photographs were cropped to the same dimension, showing only the tooth structure and avoiding other structures, which can bias the study. All the pictures segregated and were put in separate folders for each patient and were numbered 1-20. After cropping the images, all the three photographs of a single subject were put in a single powerpoint slide along with the extra-oral smile photograph and the photos were marked as A, B, C (Figure 2 - see PDF). The order of labelling and arrangement of intra-oral photos within each slide was randomized for each of the patients to avoid any bias. A powerpoint file consisting of twenty slides was made. These powerpoint slides were shown on an iPad to 52 well experienced orthodontists with clinical experience of more than ten years. All the participants in the study were aware about how to identify barrel distortion. They were then asked to rate which among the three photos in each slide had the least amount of distortion based on the extra-oral reference photograph. The responses were recorded on an excel sheet and descriptive analysis was done.

## Results and Discussion:

A total of 52 responses were collected from the orthodontists. The data collected has been shown in (Table 1 - see PDF). Among the 52 orthodontists, 41(79%) of them preferred intraoral pictures taken at a focal length of 35mm. 10(19%) of them preferred the ones taken at a focal length of 55mm and only 1(2%) orthodontist preferred the intraoral frontal picture taken at 18mm. In this study we evaluated the effects of varying focal lengths on the distortion of the intra oral images. The results show that, the majority (79%) of orthodontists preferred the intraoral images taken at a focal length of 35mm and 19% orthodontists preferred the images taken at 55mm. At a focal length of 35 mm, the optimum amount of necessary structures was visible with the least amount of barrel distortion. At 55mm, the posterior region appeared mildly distorted but still acceptable, as there was no gross distortion. The intraoral image seems to be most distorted at 18 mm focal length as only 1 (2%) among the 52 orthodontists preferred the photo (Figure 3 - see PDF). According to McKeown et al. the distortion of images is not because of the focal length, but because of the distance at which the lens is held from the subject. [5] In this study, the images were taken at different distances for different focal lengths. For a focal length of 18 mm, the lens was held much closer to the subject and for 55 mm, the lens was held away from the subject to get the whole field of interest within the frame. The distance between the lens and the object could not be standardized as the lighting of the images and field of interest became compromised as the focal length varied.  The art of taking digital photographs involves a period of learning curve, which also requires a certain basic knowledge on how a digital photograph works. [8] Proper documentation is essential for orthodontic treatments, and DDP (Digital Dental Photography) is a fundamental and widely preferred component of clinical documentation. [9,10,11] Until the intervention of digital photography, the images taken by conventional cameras could only be visible after photographic processing. In orthodontic and dentofacial treatments the patient's intraoral and extraoral appearance may change, hence high-quality photographs are mandatory for documenting these details. While taking intraoral photographs, optical distortion is a common error, which produces a barrel like effect due to the improper focal length setting. Most lenses produce some type of optical distortion, but the wide-angle lenses show pronounced barrel distortion in the images. [12] Barrel distortion describes a type of distortion wherein lines that are straight in real life appear to curve inwards (like the walls of a barrel). A good way to check for barrel distortion is to look for parallel lines in the area we are shooting and see if the lines appear parallel in our image. [13] Barrel distortion is created by the curved shape of the lens. Due to the curved lens, the center of the photo is magnified slightly more than the edges. That makes straight lines appear to curve around the edge of the image. [14] As distortion is caused by the effects of the lens, one of the ways to correct barrel lens distortion in-camera is to use a special "tilt and shift" lens, which is designed for architectural purposes. However, these lenses are costly and may not be affordable by most clinicians. [15] A basic camera and the standard 18-55mm lens were evaluated for distortion. In future, studies could evaluate the distortion of the images while using various cameras and lenses.
